# Large scale dog population demography, dog management and bite risk factors analysis: A crucial step towards rabies control in Cambodia

**DOI:** 10.1371/journal.pone.0254192

**Published:** 2021-07-08

**Authors:** Véronique Chevalier, Holl Davun, Sopheak Sorn, Pitou Ly, Vutha Pov, Sowath Ly

**Affiliations:** 1 International Center of Research in Agriculture for Development (CIRAD), UMR ASTRE, Montpellier, France; 2 ASTRE, University of Montpellier, CIRAD, INRA, Phnom Penh, Cambodia; 3 Epidemiology Unit, Institut Pasteur du Cambodge, Phnom Penh, Cambodia; 4 General Directorate of Animal Health and Production, Ministry of Agriculture, Forestry and Fisheries, Phnom Penh, Cambodia; 5 Royal University of Agriculture, Phnom Penh, Cambodia; National University of Singapore, SINGAPORE

## Abstract

Cambodia is a rabid-endemic country. However, data on dog population characteristics are lacking, and there is no national dog vaccination program. We implemented the first extensive door-to-door longitudinal survey in 2 Cambodian provinces, namely Kandal and Battambang, to estimate dog population demographic parameters, identify dog ownership determinants, analyze dog management practices and estimate the yearly cumulative bite incidence and associated factors. During the first session, more than 5000 dogs were recorded and identified. Data on families, dogs and cats characteristics, as well as the number of bites experienced the year before in the family, were recorded. One year later, a second session was performed in both provinces to record missing dogs and the reasons for missing. Age-specific survival rates of the dog populations were computed using Kaplan-Meier estimates. Ownership determinants and bite risk factors were identified using a negative binomial regression model. Dog trade and dog meat consumption were often reported. We estimated high dog-to-human ratios (1:3.8 in Kandal, and 1:3.3 in Battambang). The mean age of dog populations was 26.4 months in Kandal against 24.3 in Battambang, with a survival rate of 52% at 24 months in Kandal (34% only in Battambang). They were no feral dogs, but the large majority of recorded dogs were free roaming. In both provinces, the number of dogs significantly increased in families with children younger than 15, and when the head of the family was a male. The estimated yearly cumulative bite incidences were 2.3 and 3.1% in Kandal and Battambang provinces respectively, and are among the highest in the world. Our survey provides valuable data to focus information programs, parametrize transmission models and identify efficient vaccination strategies to control rabies in Cambodia in the future.

## Introduction

Every year, there are more than 50,000 human deaths globally due to rabies. Rabies is a zoonotic viral disease caused by a virus in the family Rhabdoviridae. It is a feared and dreaded disease: once clinical signs appeared, the issue is almost 100% fatal. Rabies virus is transmitted from infectious to susceptible dogs by bite, and the vast majority of human deaths worldwide are the result of bites from rabid dogs [1]. Most deaths occur in Asia [1–4]. Rabies is also a vaccine-preventable disease. Indeed, and since dogs are the main reservoir and source of infection for humans, vaccination of dogs is recognized as the most cost-effective and permanent solution to rabies prevention [5]. Rabies control is feasible because the basic reproduction number (R0), the average number of secondary infections produced by an infected individual–dogs in this case, in a fully susceptible population is generally low, and theoretical and empirical research has demonstrated that rabies can be eliminated where 70% coverage is sustained [6, 7]. Numerous recent programs have facilitated rabies control in low or mid-resource settings. However, reaching and maintaining this vaccination coverage can be challenging in these countries. Indeed, dog population turnover is a critical factor. Vaccination coverage declines as vaccinated animals die and susceptible puppies are born, or new unvaccinated animals are brought into the population. In areas where dog life expectancy is short, the fast dog population turnover accelerates vaccination coverage decline [8]. Comprehensiveness of vaccination campaigns is another critical point. A high proportions of free roaming dogs, or owned but aggressive dogs may result in a substantial fraction of unvaccinated dogs [9]. These unvaccinated dogs may create virus persistence pockets and jeopardize control efforts. Vaccination campaigns should, therefore, seek to achieve not just high coverage, but homogeneously high coverage [10]. Dogs are man’s commensal: their life expectancy and their accessibility are strongly linked with dog/human interactions, and the way human take care of them, or not. To settle adapted and efficient dog vaccination strategies that guarantee sufficient and sustained vaccination coverage in domestic dog populations, an in-depth knowledge of the dog populations as well as an understanding of the relationships dogs have with human is crucial [11].

Cambodia is a rabid-endemic country. The first report of rabies and vaccination in dogs has been published in 1936, and mentions a high frequency of rabies cases in carnivores and ruminants in the Kep province [12]. Nowadays, there is no official reporting of rabies human cases, but the last number of estimated deaths was 810 per year in 2007 [13]. With India where the estimated incidence was 2–3 cases per 100,000, this was among the highest published figures in Asia [14]. However, data on dog population characteristics are lacking, and there is no national dog vaccination plan in Cambodia. The only control tool existing for bitten people is the Post-Exposure-Prophylaxis (PEP), provided by five non-private entities: Institut Pasteur du Cambodge (IPC) vaccination centers in Phnom Penh, Battambang and Kampong Cham, Angkor Hospital for Children (AHC) in Siem Reap and National Institutes of Public Health (NIPH) clinic in Phnom Penh. Some private clinics also provide rabies vaccine throughout the country. However, availability, quality, and cost of PEP at these private clinics remain largely unknown [15].

The present survey is a contribution to the development of rabies control strategies in dogs in Cambodia. The goals were (i) to provide updated knowledge as prerequisite to design a dog vaccination strategy adapted to the Cambodian context, *i*.*e*. to characterize dog populations of 2 Cambodian provinces, namely Kandal and Battambang, to measure demographic parameters of the studied dog populations, to identify dog ownership determinants and analyze dog management practices, and (ii) to estimate bite incidence and associated factors to better adapt and focus information and prevention actions.

## Methodology

### Study areas

**Fig 1:**
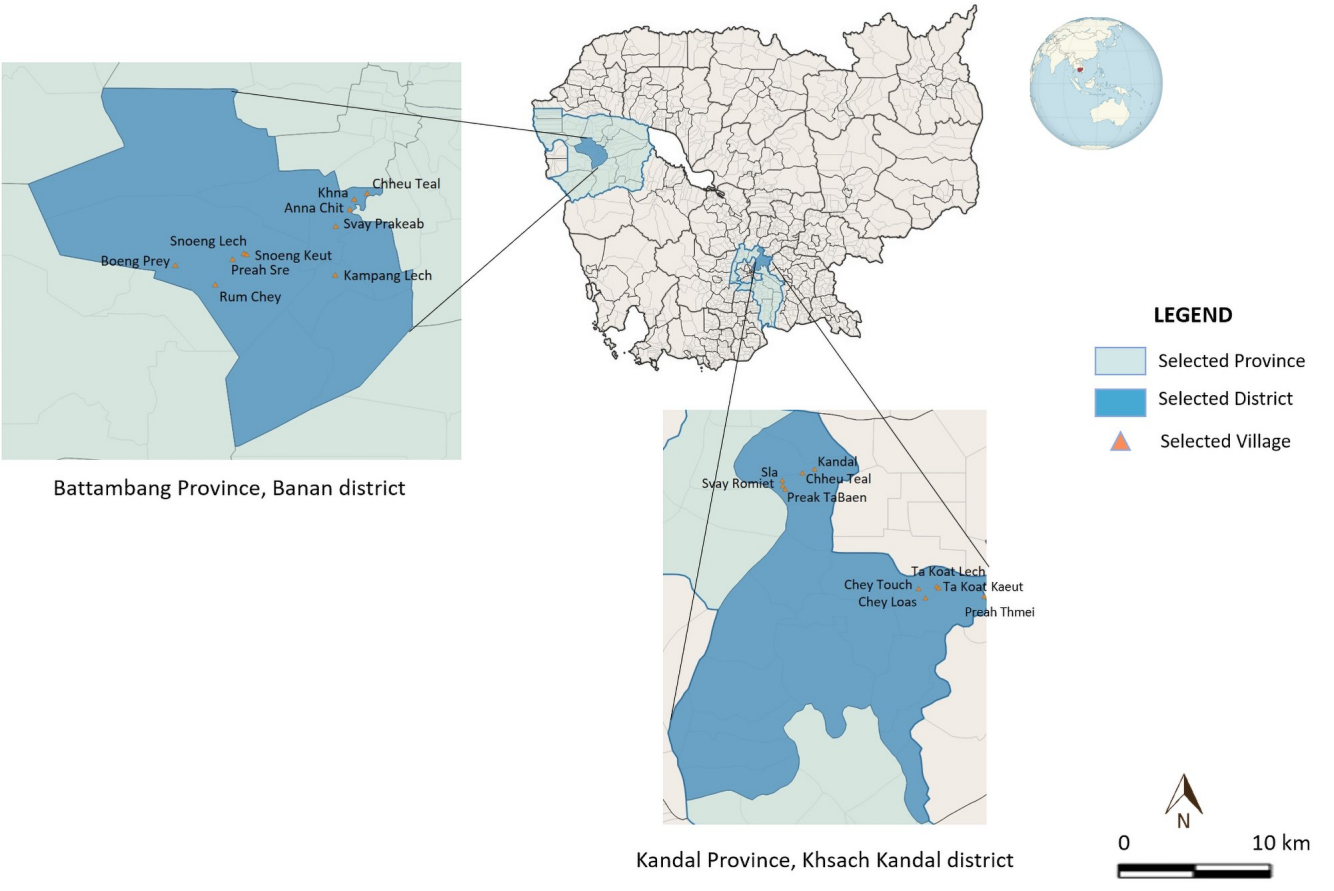
Localization of the study sites, Kandal and Battambang provinces, Cambodia 2017–2018.

Two contrasted provinces, Kandal and Battambang, were purposively selected to implement our survey. Kandal province is surrounding Phnom Penh, the capital of Cambodia, and encompassing 11 districts. Covering a 3,179 km^2^ surface area, this province consists of a typical plain wet area, dominated by cultivated fields and a mosaic of rural and peri-urban areas. Human density is 376 hab/km^2^. Most people of the province are being employed in garment and footwear factories, or handicrafts. Transportation infrastructures are well developed and the province is considered as a central base for business and trade in Cambodia [16]. In this province, the Ksach Kandal district was randomly chosen among the 11 existing districts. Then, in this district, 10 villages were conveniently chosen according to their estimated number of households (given logistical and financial constraints, we estimated that a total number of households <300 would allow a comprehensive door-to-door survey survey) and their accessibility. These villages were close to each other and located in an area dominated by rice fields. The second selected province, Battambang, is the fifth largest province of Cambodia and is located in the far northwest of the country. It is mainly a rural area, with a surface of 11702 km^2^ and a human density of 84 hab/km^2^ [16]. In 2017, Institut Pasteur du Cambodge (IPC), supported by the Ministry of Health, the Ministry of Agriculture, Forestry and Fisheries, and the Ministry of Education, Youth and Sports of Cambodia as well as the Occitanie Region (France), and in collaboration with the International Center of Research in Agronomy for Development (CIRAD), launched a program to help controlling rabies in this province. Banan district was randomly chosen among the 13 existing districts. Assuming that the dog-to-human ratio and the management practices may depend on the environment and some development metrics, we randomly selected 2 communes within the district: the Banan commune, a peri-urban area located at the close vicinity of Battambang city, and the Snoeng commune, a rural area located 20km from Battambang. Then 5 villages were randomly chosen in each commune (Fig 1).

In each village, a preliminary mission was conducted to meet the heads of the villages, explain the goals and the methodology of the survey. We recorded the number of households, the population size based on the chief knowledge or official census, and a map of the structure of each village. A verbal consent was obtained from each head of all villages included in the survey.

### Census and interviews

In March 2017 in Kandal and February 2018 in Battambang, a first door-to-door visit was conducted in each selected village (S1). During these visits, heads of households and individuals bitten by dogs or cats when they were present, were interviewed by trained interviewers. Before each interview, goals and methodology of the survey were presented again and a verbal consent was obtained from each interviewee. Three standardized questionnaires were used, one for heads of households, one for the animal identification and follow-up and one for injured persons and if any (S1 Questionnaires). The interviews were administered in Cambodian language. In addition to the family characteristics data (age and sex of the family head, number and age of children, occupation), the following data were recorded: number of dogs and cats, age, sex, and origin of dogs (gift, purchased, adopted, found). Regarding to puppies, the management of unwanted puppies (culled, abandon, gift, or raised) as well as the preferred gender were noticed. The function of dogs in the family—family member, dog meat trade, house guarding or hunting, and the mode of confinement–kept on a leash, or free roaming, were noticed. Lastly, people were asked whether they use to consume dog meat. Each dog and cat was vaccinated against rabies with 1 dose of Rabisin^®^ vaccine and individually identified with 2 or 3 pictures with the owner and display of ID codes. The number of bite events that occurred in the previous year was recorded. In Battambang, the biting species and whether this animal belonged to the family or not was also recorded. A second session (S2) was performed in both provinces one year later, in March 2018 and 2019 respectively. During this second session, the same protocol was applied with a specific focus on missing dogs and the reasons for missing. Each household was visited again. Owners were asked to recognize and identify each of the dogs he/she owns using pictures taken the year before (S1). Each dog already recorded during S1 was recorded again if seen by the interview team or declared alive by the owner but not visible during the visit. Each missing dog was identified and the reason for missing detailed by the owner. Data were subsequently anonymized.

### Statistical analysis

#### Households and animal population general characteristics, dog demographic parameters

Chi-square tests were used to compare human-to-dog ratios and proportions of females between villages or zones.

In each province, cumulative age distribution, mean and median ages were computed for the global studied populations, as well as for each village and for each zone, i.e. rural *vs* peri-urban in Battambang, using the fitdistriplus package of R 3.6.0 software [17]. We used the Welch test to compare each village-specific age mean to the mean of the studied population in Kandal, and the mean ages of the rural and the peri-urban dog population in Battambang. According to owners, missing dogs in S2 died from illness, accident, were sold, given or disappeared without any identified reason. Chi-square tests were used to assess whether the proportion of missing males and females, and the percentage of missing dogs differed between villages or zones (Battambang only). In both provinces, the survival curves and age-specific survival rates of the studied populations were computed irrespective of missing reasons, using Kaplan-Meier estimates [18]. R survival package was used for statistical analyses [17]. The log-rank test was used to assess any difference of survival rate between sex, village, and rural and peri- urban zones in Battambang only.

#### Dog ownership

To take into account overdispersion, ownership factors were analyzed using negative binomial regression model, with the number of dogs per family as model outcome, the number of children younger than 15yrs old per family, the sex and age of the family’s head—age of the head of the family was categorized in 6 classes, *i*.*e*. <30yrs, 30–40, 40–50, 50–60, 60–70, >70, the occurrence of at least one bite event in the family in the previous year, and the village as potential explanatory variables. Occupation of the head of the family and the zone was added as an additional potential predictor in Battambang. For each province, the best model was selected using a forward/backward procedure using Akaike Information Criterion (AIC) [19].

#### Bite risk factors

The risk factors of bite events were identified using a negative binomial generalized linear model, with the number of bite events per family during the past year as outcome. Potential explanatory variables were the sex and the age of the head of the family, the number of children<15yrs, the number of dogs, the number of cats, or of pets (dogs and/or cats) if any, and the village. The best models were selected with a forward/backward procedure using AIC [19].

### Ethical statement

During this study, we followed the World Animal Health Organization (OIE) guiding principles on animal welfare included in the OIE Terrestrial Animal Health Code (https://www.oie.int/en/what-we-do/standards/codes-and-manuals/terrestrial-code-online-access/). The protocol of the survey has been approved by the General Direction of Animal Production and Health GDAPH) from of Ministry of Agriculture, Forestry and Fisheries (MAFF). All interviews were implemented by trained IPC’s officers with the supervision of GDAPH agents, local veterinary services and local village authorities. All data were anonymized.

## Results

### Households and animal population general characteristics

None of these communities had been engaged in any dog population management interventions by local authorities or animal welfare organizations. Furthermore and according to owner declarations, none of the recorded dogs or cats in Kandal and Battambang were vaccinated or neutred when starting the survey. Recorded data are provided in S1 Datasets.

In Kandal, 1723 over 1908 of the declared households, *i*.*e*. a household participation rate of 90.1% (95%CI 89–92), and representing 8404 individuals, were incorporated in the survey. Mean age of the respondent population was 46.8yrs (sd = 14.7). General information on dog and cat populations are provided in S1.1 Table. As a whole, 2205 dogs were identified in Kandal, with 1001 males for 1204 females. The proportion of females, ranging from 34% to 61%, differed significantly between villages (p-value < 10^−5^). The global estimated dog-to-human ratio was 1:3.8 with large variations between villages (1:2.9 to 1:12.6). Four hundred forty cats were recorded, with a global cat-to-human ratio of 1:19.1, ranging from 1:16.2 to 1.35.4, and a cat-to-dog ratio of 1:5.0. A large proportion of recorded dogs and cats—92.2% and 87.5% respectively, could be vaccinated against rabies during S1.

In Battambang, 2082 families, representing 71.8% of the declared families and 9797 persons, were interviewed. Mean age of the respondent population was 47.7 (sd = 14.9). General information on dog and cat populations are provided in S1.2 Table. As a whole, 3010 dogs were recorded, from which 1431 were females (female-to-male ratio: 1:1.1). The proportion of females differed between villages (p-value = 0.03). The estimated dog-to-human ratio was 1:3.3, ranging from 1:2.4 to 1:6.2 in villages (p-value < 10^−10^). Highest ratios were observed in the rural villages. One thousand forty three cats were recorded, with a global cat-to-human ratio of 1:9.4 ranging from 1:6.9 to 1:11.9 and a global cat-to-dog ratio of 1:2.9. Seventy seven percent of identified dogs (2315/3010) and 73% (765/1043) of recorded cats were vaccinated against rabies during S1.

### Demographic parameters

**Fig 2:**
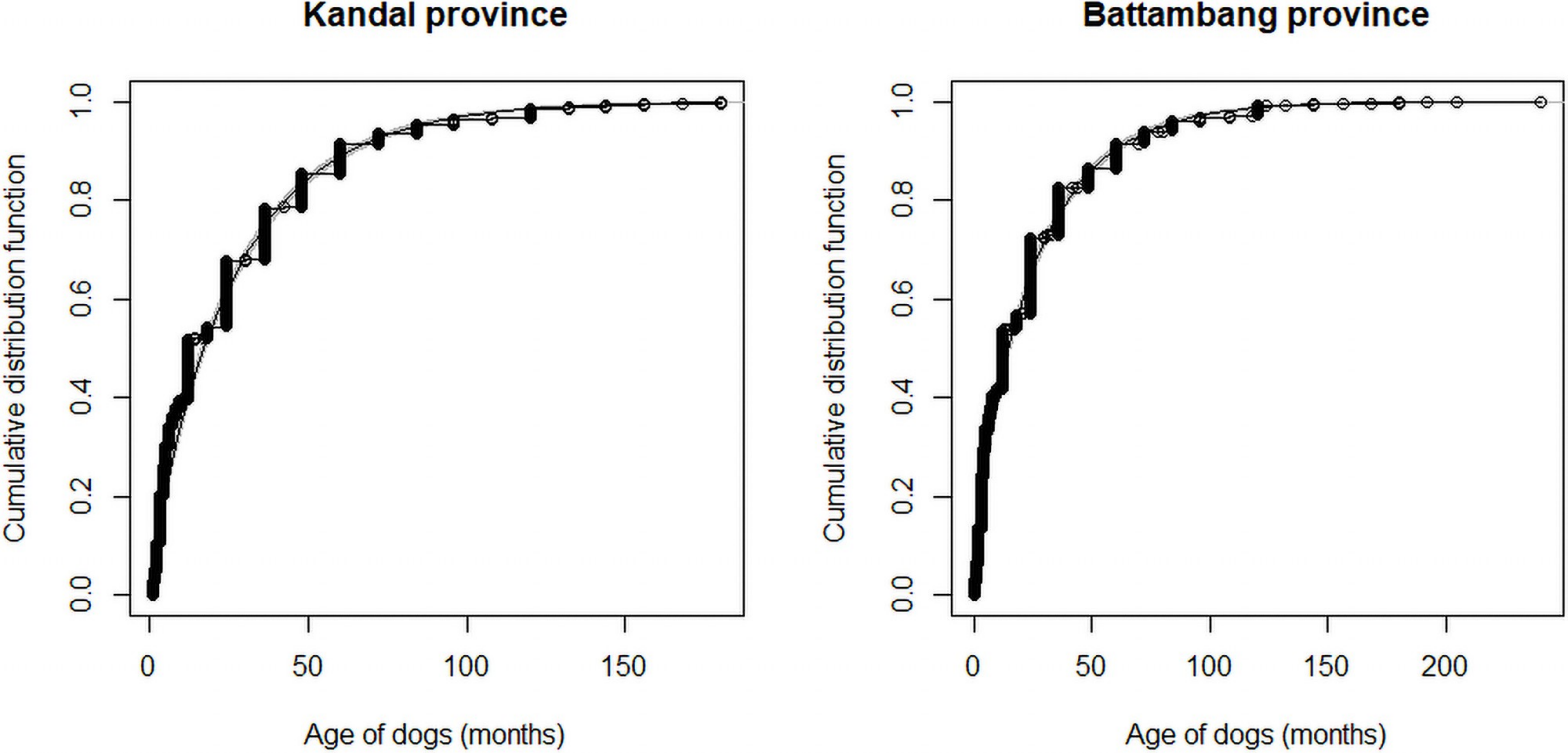
Cumulative distribution curves of dog’s ages of Kandal and Battambang provinces, Cambodia 2017–2018.

In Kandal, 20 dogs with missing ages in S1 were discarded from the dataset used for age analyses. Among the 2185 dogs included in the analyses, 1194 were females (54.6%). Cumulative distribution curves of dog’s ages of Kandal and Battambang provinces are provided in Fig 2. The Kandal studied dog population was young, with an estimated mean age of 26.4 months (sd = 30.6), a median age of 12 months, and dog ages ranging from 1 to 180 months. The estimated mean and median ages were 26.8 (sd = 28.8) and 17.5 for the female population and 26 (sd = 32.6), and 12 for the male population respectively. Cumulative distributions of dog’s ages of each village are provided in S1 Fig. Village-specific age’s means ranged from 18.3 to 39.8 months. Dog populations of Ta Koat Lech, Ta Koat Kaeut, Preak Thmei and Chheu Teal and in a lesser extent Chey Touch and Sway Somiet were significantly younger than the global one (W-test p-value< 10^−3^) (S1.3 Table).

**Fig 3:**
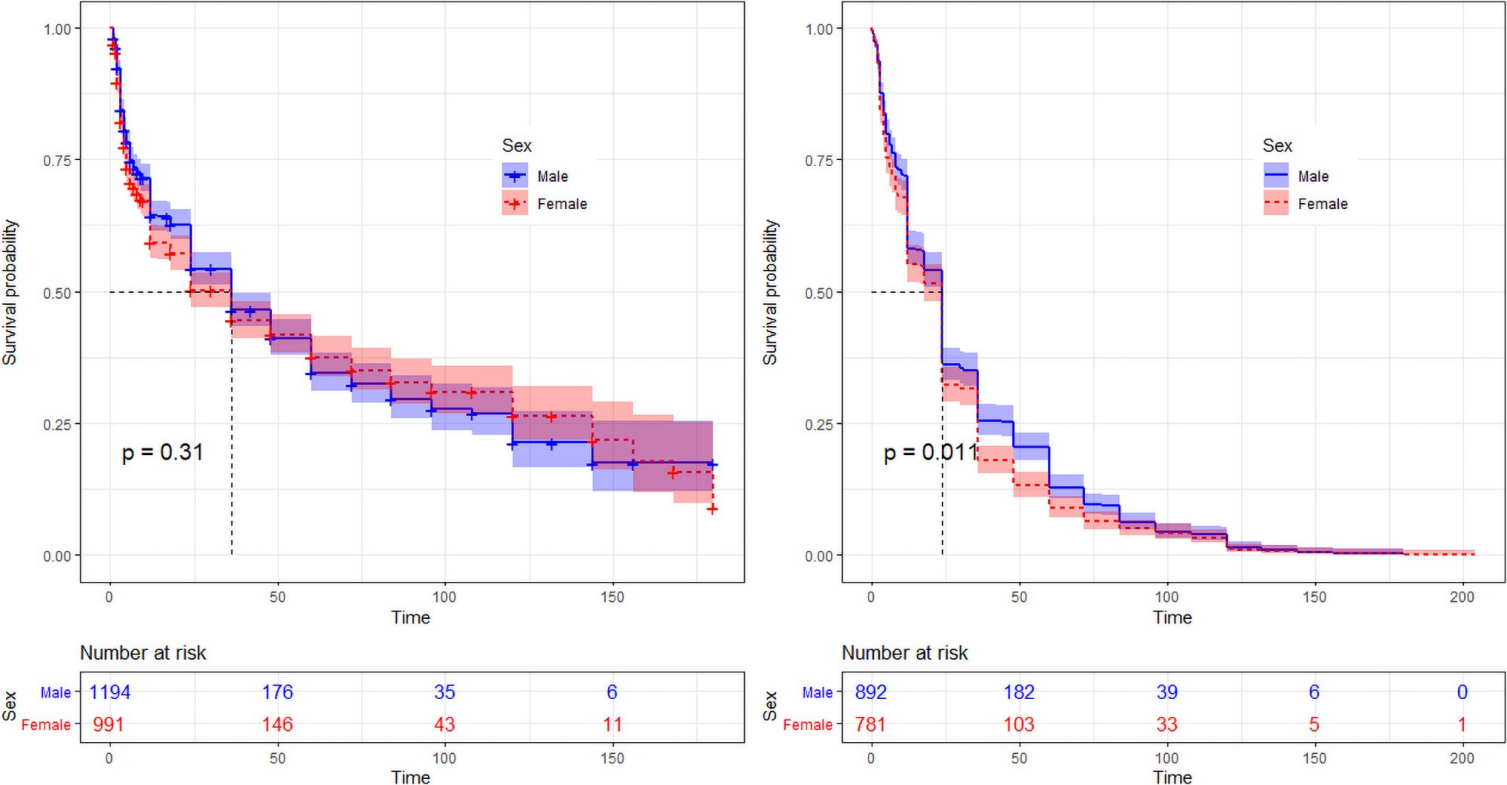
Survival probability curves of dogs per sex, in Kandal (left) and Battambang (right) provinces, Cambodia 2017–2018.

**Fig 4:**
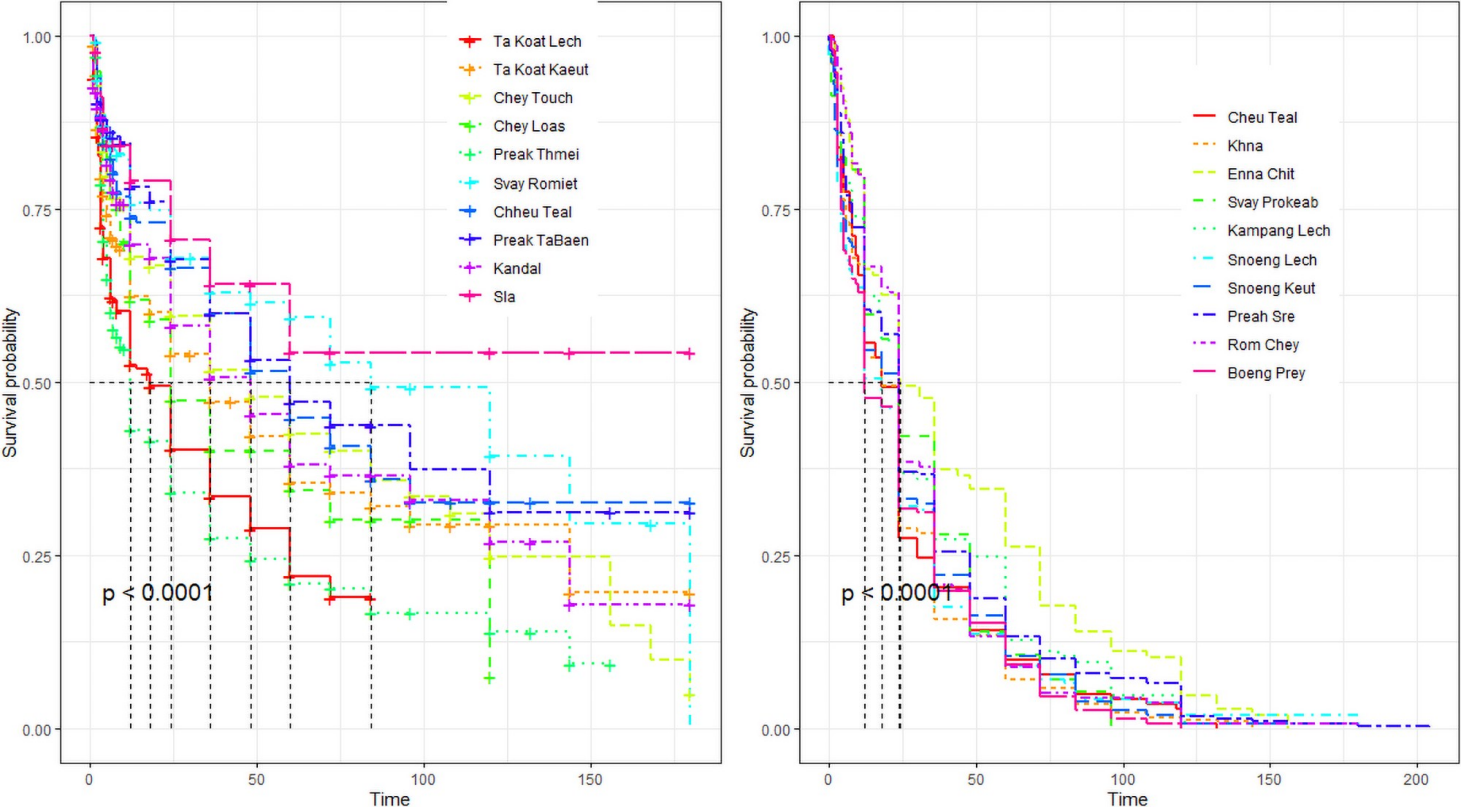
Survival probability curves of dogs per villages, in Kandal (left) and Battambang (right) provinces, Cambodia 2017–2018.

Each household visited during S1 was visited again during S2. Among the 2185 dogs recorded during S1 and incorporated in the survival analysis, 1013 (46.3%; 95%CI 44.2–48.3) were still alive, present during the visit and could be formally identified again. There was not significant difference between missing males (537/1172) and females (635/1172) proportions (p = 0.7) (S1.4 Table). Percentages of missing dogs significantly differed between villages, ranging from 34.8 to 80.1% (p<10^−5^). Among dogs identified in S1, 557 (47.5%; 95%CI 44.6–50.4) died of illness, 205 (17.5%; 95%CI 15.4–19.8) were sold, 209 (17.8%; 95%CI 15.7–20.2) disappeared, 64 (5.5%; 95%CI 4.3–7.0) were culled, 67 (5.7%; 95%CI 4.5–7.2) were given, and 70 (6.0%; 95%CI 4.7–7.5) died from road accident. According to the Kaplan-Meier estimates, the survival rate of the Kandal survey population at 12 months was 62%, 52% at 24 months, 46% at 36 months, and 41% at 48 months (S1.5 Table). The global survival rate was equivalent in both sexes (log- rank test p-value = 0.31; Fig 3), but different between villages (log-rank test p-value = 10^−4^; Fig 4). According to these estimates, the village-specific survival rates ranged from 43 to 79% at 12 months, from 34 to 71% at 24 months, 24 to 61% at 48 months and 19 to 53% at 72 months.

Similarly to Kandal, the studied dog population of Battambang was young, with an estimated age mean of 24.3 months (sd = 29.4), a median age of 12 months, and dog’s ages ranging from 2 weeks to 204 months (Fig 2). The mean and median ages were 23.4 (sd = 28.5) and 12 for the female population and 25.4 (sd = 30.2), and 12 for the male population respectively. Cumulative distributions of dog’s ages of the rural and peri-urban zones are provided in S2 Fig. On average, the rural population (n = 2146; mean age = 22.8 months) was significantly younger (p-value < 10^−4^) than the peri-urban population (n = 864; mean age = 27.8 months).

During S2, over the 2082 families initially interviewed, 646 could not be visited due to logistical constraints or reluctance of owners to participate again to the survey. Among 3010 dogs identified during S1, 1673 dogs could thus be incorporated in the survival analysis. Among these 1673 dogs identified during S1, 1169 (69.9%; 95%CI 67.6–72.1) were still alive, present during the interview and could be formally re-identified during S2. Among missing dogs, 265 were males, 239 were females. The proportion of missing dogs ranged from 18.7 to 38.2% and significantly differed between villages (p = 2.10^−3^) (S1.4 Table). Three hundred and eighteen missing dogs (63.1%; 95%CI 58.7–67.3) died from disease, 50 (9.9%; 95%CI 7.5–12.9) were sold, 49 (9.7%; 95%CI 7.3–12.7) were adopted, 39 (7.7%; 95%CI 5.6–10.4) disappeared, 31 (6.2%; 95%CI 4.3–8.7) died from road accident, 17(3.4%; 95%CI 2.0–5.3) were culled. According to the Kaplan-Meier estimates, the survival rate of the Battambang dog population at 12 months was 57%, 34% at 24 months, 22% at 36 months, and 17% at 48 months (S1.5 Table). The survival rate of the whole studied population was higher for males (log-rank test p-value = 0.01; Fig 3), and significantly differed between villages (p<10^−4^; Fig 4); the survival rate of the rural population was lower than for the peri-urban one (log-rank test: p-value = 0.01). Age-specific survival rates were also lower in Battambang than in Kandal, with 73% *vs* 76% in Kandal at 6 months, then 57% *vs* 62% at 12 months, 34% *vs* 52% at 24 months, 22% *vs* 46% at 36 months and 17% *vs* 41% at 48 months (S1.5 Table).

#### Dog ownership

In Kandal, over the 1723 families interviewed during S1, 1017 (59%; 95%CI 56.7–61.4) declared owning at least 1 dog. The mean number of dogs per family was 1.3 (sd = 1.6), and 2.2 (sd = 1.5) for families having at least 1 dog. The maximum number of dogs per family was 14 (S1.6 Table). The best regression model incorporated the number of children younger than 15yrs, the sex and age of the family’s head, the occurrence of at least one bite event during the previous year and the village as explanatory variables. According to this model (Table 1), families were more prone to own dogs when the head of the family was a male (p<10^−3^). Head of families between 50 and 60 years old had more dogs than younger people (p = 0.03). The number of dogs per family significantly increased with the number of children younger than 15yrs (p<10^−10^), but decreased when bite events occurred in the family during the previous year (p<10^−10^). Ownership differed between villages, with a smaller number of dogs per family in Chey Loas, Svay Romiet, Preak TaBaen and Sla villages, compared to Ta Koat Kaeut.

**Table 1:** Dog ownership determinants in Kandal province: Results of the negative binomial regression model with the number of dogs per family as model outcome, the number of children younger than 15yrs, the sex and age of the family’s head, occurrence of at least one bite event during the previous year and the village as explanatory variable.

		Estimate	Incidence rate ratio (CI 2.5%-97.5%)	p- value
	Intercept	0.0152	1.02 (0.80–1.28)	0.90
**Sex**	female	ref	ref	ref
	**male**	**0.1729**	**1.19 (1.07–1.32)**	**<10**^**−3**^
**Age**	<30yrs	ref	ref	ref
	30–40	-0.0270	0.97 (0.80–1.19)	0.79
	40–50	0.1537	1.17 (0.95–1.43)	0.14
	**50–60**	**0.2301**	**1.26 (1.03–1.55)**	**0.03**
	60–70	0.20077	1.22 (0.98–1.53)	0.07
	>70	0.09826	1.10 (0.85–1.43)	0.46
**Village**	Ta Koat Lech	ref	ref	ref
	Ta Koat Kaeut	0.0225	1.02 (0.83–1.27)	0.84
	Chey Touch	-0.0842	0.92 (0.74–1.15)	0.46
	**Chey Loas**	**-0.3175**	**0.73 (0.56–0.95)**	**0.02**
	Preak Thmei	0.0280	1.03 (0.83–1.27)	0.80
	**Svay Romiet**	**-0.6063**	**0.54 (0.42–0.71)**	**<10**^**−5**^
	Chheu Teal	0.2535	1.29 (0.97–1.70)	0.07
	**Preak TaBaen**	**-0.6858**	**0.50 (0.39–0.65)**	**<10**^**−6**^
	Kandal	0.19288	1.21 (0.96–1.54)	0.11
	**Sla**	**-1.4688**	**0.23 (0.16–0.33)**	**<10**^**−10**^
	**Nb of children <15y**	**0.1354**	**1.15 (1.09–1.20)**	**<10**^**−10**^
**Bite event**	0	ref	ref	ref
	**1**	**-0.7832**	**0.46 (0.36–0.57)**	**<10**^**−10**^
	**2**	**-1.2366**	**0.29**	**6.10**^**−3**^

In Battambang, 1260 of families (60.5%) declared owning at least 1 dog (S1.7 Table (a,b)). The mean number of dogs per family of the whole studied population was 1.4 (sd = 1.8), and 2.4 (sd = 1.7) for families having at least 1 dog. The maximum number of dogs was 8 in the peri-urban zone against 14 in the rural zone. The best model incorporated the age of the owner, his/her sex, the village and the number of children younger than 15yrs. According to this model, and similarly to Kandal, the number of dogs per family increased with the number of children <15yrs (p = 4.10^−3^), when the head of the family was a male (p<10^−5^), and for owners being between 40 and 60yrs old (p = 2.10^−4^ and 3.10^−4^). Compared to Annachit, located in the peri-urban zone, the number of dogs per family increased in Boeng Prey (p<10^−5^), Preah Sre (p<10^−5^), Rum Chey (p = 0.02) and Snoeng Keut (p = 3.10^−3^), all located in the rural zone (Table 2). Neither owner’s occupation nor the zone or the occurrence of bite events in the previous year influenced the dog ownership.

**Table 2:** Dog ownership determinants in Battambang province: Results of the negative binomial regression model with the number of dogs per family as model outcome, the number of children younger than 15yrs old, the sex and age of the family’s head and the village as explanatory variable.

		Estimate	Incidence rate ratio (CI 2.5%-97.5%)	p- value
	Intercept	-0.2240	0.80 (0.63–1.01)	0.06
**Sex**	female	ref	ref	ref
	**male**	**0.2534**	**1.29 (1.16–1.43)**	**<10–5**
**Age**	<30yrs	ref	ref	ref
	30–40	0.12	0.13 (0.94–1.36)	0.18
	**40–50**	**0.3396**	**1.40 (0.16–1.70)**	**3.10**^**−4**^
	**50–60**	**0.3353**	**1.40 (1.17–1.68)**	**2.10**^**−4**^
	60–70	0.1287	1.31 (0.93–1.39)	0.21
	>70	0.06	1.07 (0.84–1.35)	0.58
**Village**	Anna Chit	ref	ref	ref
	**Boeng Prey**	**0.4906**	**1.63 (1.33–2.10)**	**<10**^**−5**^
	**Chheu Teal**	**-0.39934**	**0.67 (0.52–1.12)**	**3.10**^**−3**^
	Kampang Lech	-0.23182	0.79 (0.56–1.12)	0.2
	Khna	0.0199	1.02 (0.79–1.32)	0.8
	**Preah Sre**	**0.5245**	**1.70 (1.34–2.13)**	**<10**^**−5**^
	Rum Chey	0.2694	1.31 (1.04–1.65)	0.02
	**Snoeng Keut**	**0.3264**	**1.39 (1.12–1.72)**	**3.10**^**−3**^
	Snoeng Lech	-0.189	0.83 (0.64–1.08)	0.1
	Svay Prakeab	0.1472	1.16 (0.90–1.50)	0.26
	**Number of children <15yrs**	**0.0596**	**1.06 (1.02–1.11)**	**5.10**^**3**^

### Dog management

Results are provided in S1.8 Table. The function of dogs in the family significantly differed between Kandal and Battambang (p<10^−5^). The majority of families, 78.1% (95%CI 75.4–80.6) in Kandal and 98.1% (95%CI 97.2–98.8) in Battambang, raised dogs to protect the house. However, 20.4% of families in Kandal (95%CI 17.9–23.0) considered dogs as “family member”, against 1.4% (95%CI 0.9–2.3) only in Battambang. In both provinces, a low percentage of families declared raising dogs for business and/or dog meat trade: 1.4% (95%CI 0.8–2.3) in Kandal and 0.2% (95%CI 0.06–0.80) in Battambang. However, data collected during this survey clearly showed that dog trade exists: in Kandal, 79.2% (95%CI 77.2–81.2) of the 1723 interviewed families, and 27.5% (95%CI 24.8–30.4) of families having dogs, declared selling and/or buying dogs. In Battambang, only 18.2% (95%CI 16.1–20.4) of families with dogs declared trading dogs, against 13.4% (95%CI 14.0–17.5) of the overall number of interviewed families. Only 2 families, in both Kandal and Battambang used dogs for hunting.

According to our data, dog meat consumption was more frequent in Battambang than in Kandal (p< 10^−10^): 58.6% (95%CI 56.4–60.8) of all interviewed families, and 64.4% (95%CI 61.7–67.1) of families having dogs in Battambang declared consuming dog meat from time to time, against 23.4% (95%CI 21.5–25.5) of the Kandal studied population, and 28.7% (95%CI 25.8–31.4) of families having dogs. Consumed dogs belong to the family or are purchased from either traders or restaurants.

The majority of dogs were free-roaming in both provinces: only 6 families (0.6%; 95%CI 0.24–1.30) used to confine their dogs in Kandal and 13 in Battambang (1%; 95%CI 0.60–1.80). Most of families, *i*.*e* 98.3% (CI95% 97.3–99.0) and 99.5% (CI95% 98.9–99.8) in Kandal and Battambang respectively, used to keep puppies and raise them. Other families use to give them to neighbors or to family members. Remaining families declared selling, culling or abandoning the unwanted puppies. Our results showed a clear preference for males in Battambang compared to Kandal (p-value <10^−6^). Regarding to dog’s origins, 43.8% (95%CI 41.7–45.9) and 50.2% of recorded dogs during S1 (95%CI 48.4–52.1) in Kandal and Battambang respectively were born in the family; 30.7% (95%CI 28.8–32.7) were given in Kandal and 45.2% (95%CI 43.4–47.0) in Battambang. Around a quarter (23%; 95%CI 21.3–24.9) of recorded dogs were purchased in Kandal against only 1.4% (95%CI 0.9–1.8) in Battambang. A very low fraction of recorded dogs were found, *i*.*e* 2.4% and 3.2% in Kandal and Battambang respectively.

#### Bite incidence and risk factors

In Kandal, 196 bite events that occurred in the previous year were recorded for 8404 people included in the survey, *i*.*e*. a yearly bite incidence of 2,3% (CI95% 2.0–2.7). Fourteen families declared 2 bite events. 58.7% of bites occurred in families without dogs. Yearly bite incidence estimations per village are provided in S1.1 Table. Strong differences are observed between villages, with bite incidence ranging from 0.9 to 4.2%. This result is confirmed by the best statistical model which incorporates the number of children <15yrs in the family, the village and the number of animals (dog or cats) owned by families. According to this model, the number of bite events recorded per family and per year significantly increased with the number of young children (p<10^−4^). As expected, the number of animals owned by families has no effect on the yearly bite incidence (Table 3).

**Table 3:** Identification of bite risk factors on the interviewed population (n = 8404) in Kandal province: Results of the negative binomial generalized linear model with the number of bite events per family and per year as output, and the village, the number of children <15yrs in the family and the number of animals (dog and/or cat) owned by the family as explanatory variables.

		Estimate	OR (CI 2.5% -97.5%)	p value
	Intercept	-2.08	0.13 (0.08–0.18)	<10^−10^
	Number of owned dog and/or cats	0.06	1.06 (0.98–1.14)	0.12
**Village**	Ta Koat Lech	Ref	ref	ref
	Ta Koat Kaeut	0.01	1.01 (0.64–1.63)	0.96
	Chey Touch	-0.39	0.67 (0.40–1.14)	0.14
	**Chey Loas**	**-1.44**	**0.23 (0.09–0.54)**	**10**^**−3**^
	**Preak Thmei**	**-0.75**	**0.47 (0.28–0.80)**	**5.10**^**−3**^
	Svay Romiet	-0.83	0.44 (0.22–0.83)	0.01
	Chheu Teal	-0.65	0.52 (0.22–1.10)	0.11
	Preak TaBaen	-0.71	0.49 (0.26–0.90)	0.02
	**Kandal**	**-1.05**	**0.35 (0.16–0.70)**	**5.10**^**−3**^
	Sla	-0.91	0.40 (0.19–0.81)	0.01
	**Nb of children <15yrs**	**0.20**	**1.22 (1.11–1.32)**	**10**^**−5**^

In Battambang, 304 bite events were declared for 9797 people included in the survey, *i*.*e*. a yearly bite incidence of 3.1% (CI95% 2.8–3.5). Thirty two families declared 2 bite events, and 4 families 3 bites events. Eighty four bites (27.6%; CI95% 22.7–33.1) were caused by cats, 183 (60.2%; CI95% 54.5–65.7) by animals that did not belong to the family (162 dogs, 21 cats), and 121 (39.8%; CI95% 34.3–45.6) bites were recorded in families without dogs. The village-specific incidence rate ranged from 2.2 to 4.9% (S1.2 Table). According to the best model that incorporates the number of children younger than 15yrs, the number of cats per family and the village, families owning cats experienced more bite events during the year (p = 4.10^−4^). The number of bite events also increased with the number of children younger than 15yrs (p<10^−5^). The risk of being bitten, at the family level, was also linked to the village: families living in Boeng Prey appeared less at risk than other villages, and in particular compared to Anna Chit, the reference village (Table 4).

**Table 4:** Identification of bite risk factors on the interviewed population (n = 9797) in Battambang province: Results of the negative binomial generalized linear model with the number of bite events per family per year as output and the village, the number of children <15yrs in the family and the number of cats owned by the family as explanatory variables.

		Estimate	OR (CI 2.5% -97.5%)	p value
	**Intercept**	**0.12**	**1.13 (1.06–1.20)**	**2.10**^**−4**^
	**Number of owned cats**	**0.03**	**1.03 (1.01–1.05)**	**4.10**^**−4**^
**Village**	Anna Chit	ref	ref	ref
	**Boeng Prey**	**-0.10**	**0.90 (0.84–0.97)**	**5.10**^**−3**^
	**Chheu Teal**	**-0.09**	**0.92 (0.85–0.99)**	**0.04**
	Kampang Lech	0.02	1.02 (0.92–1.14)	0.71
	Khna	0.05	0.96 (0.88–1.04)	0.30
	Prey Sre	-0.05	0.95 (0.88–1.04)	0.23
	Rum Chey	-0.01	0.99 (0.91–1.07)	0.78
	Snoeng Keut	-0.004	0.99 (0.93–1.07)	0.92
	Snoeng Lech	-0.08	0.92 (0.85–1.00)	0.05
	Svay Prakeab	-0.08	0.92 (0.84–1.00)	0.06
	**Nb of children <15yrs**	**0.03**	**1.031 (1.02–1.05)**	**<10**^**−5**^

## Discussion

Cambodia is a rabid-endemic country, with a high estimated yearly burden on human health [13]. However, data documenting the dog population characteristics, as well as the human/dog relationships that are essential to design future vaccination campaigns are lacking. This paper presents the results of the first large scale dog population door-to-door survey in Cambodia, with more than 5000 dogs recorded, and provides valuable data to design information programs, parametrize transmission models and identify efficient vaccination strategies to control rabies in this country in the future.

Many surveys have been implemented around the world to estimate dog-to-human ratios as basic but fundamental knowledge needed to plan massive dog vaccination programs against rabies. These ratios vary from 91 dogs for every 100 people in the Philippines [20] to 2 dogs for every 100 people in urban areas of Zambia [21]. In Chile, the human-to-dog ratio ranged from 5.2 to 6.2 in cities, from 2.3 to 5.3 in towns, and from 1.1 to 2.1 in rural areas [22]. However for most of studied populations, this ratio stands between 1 and 3.3 dogs per 100 people [23]. In Cambodia, a first census was performed in 7 provinces between 2005 and 2007: a total of 2670 owned dogs were recorded for 8269 individuals surveyed yielding a ratio of 1 dog to 3.1 humans (95%CI 1∶3.0–1∶3.2) [13]. A second survey was conducted in 2007: the dog-to-human ratio was 1:4.3 in Kandal and 1:4.8 in Phnom Penh, the capital [24]. The last survey was performed in Siem Reap in 2013. The dog-to-human ratio was 1:2.8 [25]. Our estimations—1:3.8 in Kandal, and 1:3.3 in Battambang- are very close to the previous ones, suggesting a stable dog-to-human ratio over years. Even if rarely included in these kinds of survey, the recorded cat population was far from negligible, with a global cat-to-human ratio of 1: 9.4 in Battambang. As a matter of fact, from 2000 to 2016, 12 heads of biting cats over 46 (26.7%) tested positive for rabies in the frame of the passive surveillance implemented by IPC (L. Sowath, personal com), suggesting that cats probably participate to rabies burden in Cambodia, and should be incorporated in a future massive vaccination campaign in this country.

The studied dog populations appeared young with a low survival rate before the age of 2yrs. Demographic parameter values are very close to was has been estimated in other low or middle income and rabid endemic countries. In Tanzania, the mean age of the studied dog population was 2.23 years (95%CI = 2.06–2.55 / Median = 2yrs). Age-specific mortality was very high in the under one year olds (72%) and the four year olds (80%) [26]. In Kenya, 50% of the recorded dogs were 1 year old or less, and the overall mean age was 1.9 years (2.1 years for males and 1.6 years for females) [27]. Mean and media ages were respectively of 2.1 and 2 years in a rural area of Bali [28].

Even if individual identification of dogs using microchips could not be done due to logistical constraints, all dogs included in the survival analysis were strictly and systematically re-identified during S2 by owners using the 2 or 3 pictures taken of each dog with the owner or a member of the family the year before. Age of animals were given in months by owners. Since most of these animals were young, we assumed that the memory bias was reduced. We did not ask any questions about the date of the dog disappearance. The Battambang dog population was younger than the Kandal one, with an estimated survival rate of 17% at 48 months against 48% in Kandal. Within Battambang province, the rural population was younger than the peri-urban one. Illness and poor health conditions observed in Battambang, especially in the rural zone probably linked to lower economic and development metrics and poor dog management, may partially explain these differences. As a matter of fact, 20.4% of interviewed families in Kandal considered dogs as “family member”, against 1.4% only in Battambang. Furthermore 62% of dogs recorded in S1 died between S1 and S2 from illness in Battambang against 47.5% in Kandal. In both provinces, large disparities of the survival rates were observed between villages. These differences may be explained by dog trade practices, or again, by lack of attention paid to the dogs. Whatever the reasons, these differences may have consequences in terms of vaccination efficiency, should the highest survival rate be applied instead of the lowest to design the vaccination frame. Despite a global low survival rate, estimated dog-to-human ratios are comparable to what has been estimated in 2007, suggesting again that the dog population size is stable in areas of concern, the high level mortality being compensated by a high birth rate. Survival rates were computed irrespective of missing reasons. Some of missing dogs were given or sold, to be culled or to be replaced, or not, by another dog. In the case of a vaccinated population, some of these sold or culled dogs would be replaced by susceptible animals, whereas some of these dogs would simply be moved from a village to another one, alive and immune. In our survival analysis, we thus considered the worst case scenario in which, whatever the reason, each missing dog could potentially be replaced by a new susceptible dog.

Informal discussion with owners confirmed that they were no or very few feral dogs in the studied areas, but the large majority of recorded dogs were free roaming. Ownership determinants were quite similar in both provinces: the number of dogs increased in families with children younger than 15 years old, and when the head of the family was a male. Females frequently explained during the informal interview that they were frightened by dogs.

Dog movements, human-mediated or not, have an important impact on rabies spread [29, 30]. Indeed, the frequency of campaigns required to maintain vaccination coverage above a critical threshold depends on the introductions of susceptible individuals into the population by people through the acquisition of dogs born locally or from outside the population and the loss of vaccinated individuals through deaths and the relocation of dogs by people: ignoring dog movements may lead to vaccination program failure [31]. Our survey showed that dog trade was prevalent in both provinces, but apparently more frequent in Kandal than in Battambang. On the opposite, dog meat consumption seemed more frequent in Battambang. We observed discrepancies in both provinces between the low percentage of people who declared having dogs for trade (instead of guarding the house or as a family member) and people who declared selling /buying dogs from time to time. Our survey did not focus on dog trade *per se*. When people were asked about the function of the dog in the family, the modality “raising dogs for meat “was understood as a commercial and regular activity, explaining why most of people answered “no”: in both studied provinces, people do not raise dogs for business. However, opportunistic trade dog practices remain frequent: people may sell dogs when they need money or need to barter it against cooking material for instance. People also sometimes eat dog meat when they are celebrating (birth, wedding..). So, when people were asked why they owned dogs, these people spontaneously replied “to guard the house”, but when asking whether they sometimes trade dogs, a large majority of them answered yes, as if there was no relationship between the function of the dog at home, and the way they can occasionally use it when needed.

Existence of dog meat trade has already been documented in Cambodia. A survey conducted by the Four Paws non-governmental organization showed that Siem Reap province, located in the north of the country, was a ‘hot spot’ for dog sourcing, supplying the demand for dog meat in the Eastern half of the country, most notably Phnom Penh. Some of traded dogs are stolen pets. Investigation found minivans filled with dogs arriving at slaughterhouses in Kandal Province, among others, from Siem Reap daily. An estimated 3750 live dogs would be exported out of Siem Reap province every month for slaughtering. At the country level, 2–3 million dogs may be slaughtered for the trade in Cambodia annually [32]. These traded dogs probably do not participate to rabies long distance-spread since they are culled as soon as they are delivered. However, the major fraction of this highly dense dog population is free roaming before being captured and traded, and therefore may contribute to rabies endemicity in Cambodia. Furthermore, around 50% of our recorded dogs only were born in the families. The other dogs came from a neighboring village, but sometimes, and according to informal discussions the interviewers had with participants, from another district, province or even another country. Our survey did not specifically focused on dog movements. However our preliminary results suggest that dog movement patterns and drivers at local, medium and large scale in Cambodia are part of a complex socio-economic system that should be deeply understood to maximize the efficiency of rabies control programs.

According to our data, the estimated yearly bite incidence rate was 2.3 and 3.1% in Kandal and Battambang provinces respectively. An average bite incidence rate of 2.5% roughly extrapolated to the whole country–the estimated Cambodian population size is 17 millions) would imply 425 000 bites injuries per year. In 2020, around 50 000 people came to IPC PEP center to get vaccinated after being bitten (S. Ly personal com): a large proportion of bitten people probably remain untreated and there is an urgent need to estimate the number of people who have been exposed to rabies among these people. Our estimated bite incidence rates are higher than estimations provided in Phnom Penh, the capital, and Kandal provinces in 2009 (1,12% (95% CI 1.05–1.19) [24], and similar to what was found in India (17–19.6/1000) confirming that Cambodia, with Bangladesh (72.9/1000) is one of the countries experiencing the highest bite incidence rate in the world [33–35]. For comparison, the estimated annual dog bite incidence was 5.2 per 1000 person years in a rural area of Congo in 2017 [36]. Similarly to previous surveys performed in Cambodia and other countries, children represent a high-risk population in both provinces and need to be properly educated about dog bites risks [24, 28, 34, 36, 37]. In both provinces also, a large proportion of bites are caused by animals that do not belong to the family, these probably resulting from the high proportion of free roaming dogs. The bite incidence also significantly varied between villages. Underlying explanations should be further investigated to improve information and prevention actions. Cats are responsible of a significant number of bites, emphasizing again the need to include them in a future vaccination campaign against rabies.

The most critical factors that determine the effectiveness of vaccination are the level of vaccination coverage achieved and the comprehensiveness of campaigns [10]. Our initial goal was to vaccinate 100% of recorded dogs and cats. We globally had good access to dogs, with higher success rate in Kandal than in Battambang where dogs were more aggressive and difficult to handle.

Our survey provide crucial knowledge to implement rabies control strategies in dog population in Cambodia. Kandal and Battambang results are rather similar. However, they can probably not be extrapolated to the whole country. Mondolkiri or Ratanakiri provinces for instance, located in the northeastern part of the country, are characterized by large protected areas where communities still use to hunt with dogs, and where feral dog groups may be observed. In these provinces, a large proportion of dogs can not be handled even if parenteral vaccination remains the method of choice to control rabies transmission in dog populations. Oral vaccination has shown its efficacy by eliminating sylvatic rabies over almost all the European Union territory and should be considered a useful alternative [38–40]. However dog vaccination alone may not be sufficient. Inadequate knowledge of rabies, poverty and poor dog management are major obstacles to the control of rabies [25, 41]. In Cambodia, rabies control should rely on a One Health, multidisciplinary and multi-sectoral approach: besides dog vaccination, an important effort should be dedicated, first, to information and prevention, and second, to an in-depth understanding of the socio-economical context of dog ownership, in order to propose adapted rabies control measures that can be endorsed by communities and dog owners.

## Supporting information

S1 Questionnaires (PDF)

S1 Datasets (XLSX)

S1 Table (PDF)

S1 Fig Cumulative distributions of dog’s ages of per village in Kandal province, Cambodia, 2017. (TIFF)

S2 Fig Cumulative distributions of dog’s ages of the rural and peri-urban zones in Battambang. (TIFF)
