# Supplementary material for: Large scale dog population demography, dog management and bite risk factors analysis: A crucial step towards rabies control in Cambodia
Source: PLoS One. 2021 Jul 8;16(7):e0254192. doi: 10.1371/journal.pone.0254192 (PMC8266089; doi:10.1371/journal.pone.0254192)
Supplement: S1 Questionnaires — (PDF) [file pone.0254192.s001.pdf]

Supplementary information S1

Questionnaires

Household information

Household ID -DVI code

CODE:

N |_______________________________|

Respondent

E |_______________________________|

Name |____________________| Village |_______________________________|

Gender 1[_] Male 2[_] Female Commune |_______________________________|

Age |_______| (current age in year) District |_______________________________|

Province |_______________________________|

Telephone |_______________________________|

Interview date (dd/mm/yy)

Date |__________|

Number of household membe |__________|

1) Number of household members who are at risk (age < 15 y |__________|

2) Number of household members who got injuried by dog/cat during the previous |________|

Nº	ID	Name	Age	Gender
1				
2				
3				
4				
5				

Bite characteristics

Information about injuries caused by dog/cat

ID CODE: Accident Nº: Name: |______________________________|

1 form = 1 event

I. Circumstance

1. When? Date of accident |_____________| (at least month and year 9[_] Do not remember the date

2. Where? 1[_] In the village 1[_] Outside the village

II. Animal information

3. Specie? 1[_] Dog 2[_] Cat

4. Ownership? 1[_] Own dog 2[_] Other's dog 9[_] Do not know

5. Aspect of the dog? 1[_] Sick 2[_] Healthy 9[_] Do not know

III. Wound

6. Exposure Mode? 1[_] Bite 2[_] Licking 3[_] Scratching 4[_] Bite and Scratching

7. Bleeding? 1[_] Yes 0[_] No

8. Location? 1[_] Head/Neck 2[_] Upper limb 3[_] Lower limb 4[_] Body 5[_] Genital

IV. Health seeking behavior

9. Wound stuture? 1[_] Yes 0[_] No

10. Wound care?

11. Received vaccination against tetanus? 1[_] Yes 0[_] No 9[_] Do not know

12. Received vaccination against rabies? 1[_] Yes 0[_] No 9[_] Do not know

13. If yes, where?
1[_] IPC Rabies vaccination centre
2[_] NIPH
3[_] Public HC/Hospital
4[_] Private clinic
5[_] Others: |_____________|

V. Outcome at the time of interview

14. General conclusion on health status of the patient
1[_] Good health 2[_] Living with illness **>>> go to 15** 3[_] Death **>>> go to 16**

9[_] No news since |_____________|

15. If the patient lives with illness, what illness? |______________________________________|

16. If the patient died, what was the cause? |______________________________________|

Dog census and follow-up

**Total Dog or Cat** (ចំនួនសត្វសុនខ ឬ ឆ្មា សរុប)					
	**Animal (1)**	**Animal (2)**	**Animal (3)**	**Animal (4)**	**Animal (5)**
**ID**					
Name					
Gender					
Age					
BCS					
Pregnant Status	Yes , No ,	Yes ,	Yes ,	Yes ,	Yes ,
Paturition					
Origin					
Status : (1 = From owner's bitch) , (2 = Adopt from pagoda or found accidentally) , (3 = Gifted from someone) , (4 = Purchased from someone)					
Confine level					
Status : (1 =Attach inside or under the house) , (2 = Free roaming) , (3 = Haft attach haft free roaming)					
Vaccination	Yes /	Yes	Yes	Yes	Yes
Vaccine-Date	/	/	/	/	/
Vaccine-Provider					
V2. Date	[ ]	[ ]	[ ]		[ ]
V3. Date	[ ]	[ ]	[ ]		[ ]
V4. Date	[ ]	[ ]	[ ]		[ ]
V5. Date	[ ]	[ ]	[ ]		[ ]
Status : (1= Alive ; 2= Culled ; 3= Died of illness ; 4= Sold out ; 5= Disappeard ; 6= Adopted/given away; 7= Died of accident)					
