# Supplementary material for: Large scale dog population demography, dog management and bite risk factors analysis: A crucial step towards rabies control in Cambodia
Source: PLoS One. 2021 Jul 8;16(7):e0254192. doi: 10.1371/journal.pone.0254192 (PMC8266089; doi:10.1371/journal.pone.0254192)
Supplement: S1 Table — (PDF) [file pone.0254192.s003.pdf]

Tables

S1.1_Table: Interviewed population, dog and cat population, ratios and bite incidence, Kandal province, Cambodia, 2017

Village Names (GPS coord)	Population size	Total number of Households (HH)	Number of interviewed HH	Size of the interviewed population	Number of recorded dogs during S1 (%vaccinated)	Number of males	Number of females	Number of recorded cats during S1 (%vaccinated)	Observed dog to human Ratio	Observed cat to human Ratio	Number of bite events recorded in the previous year (per 100 people included in the survey)
**Ta Koat Lech** (11,72702; 105,12244)	823	185	152	692	210 (97)	88	122	33 (32)	1:3.3	1:21	28 (4.0)
**Ta Koat Kaeut** (11,72546; 105,12383)	1112	293	257	1174	383 (94)	153	230	60 (60)	1:3.1	1:19.6	49 (4.2)
**Chey Touch** (11,72547; 105,10988)	1216	219	222	1102	309 (95)	147	162	68 (61)	1:3.6	1:16.2	29 (2.6)
**Chey Loas** (11,71901; 105,11574)	780	154	135	640	141 (94)	57	84	34 (33)	1:4.5	1:18.8	6 (0.9)
**Preak Thmei** (11,70786; 105,15298)	1876	263	279	1458	446 (98)	170	276	73 (67)	1:3.3	1:20	27 (1.9)
**Svay Romiet** (11,79629; 105,01456)	828	132	158	815	132 (96)	75	57	23 (21)	1:6.2	1:35.4	13 (1.6)
**Chheu Teal** (11,80029; 105,02307)	890	143	75	363	150 (87)	75	75	20 (19)	1:4.2	1:18.2	8 (2.2)
**Preak TaBaen** (11,7906; 105,01977)	1109	202	173	839	130 (82)	74	56	65 (55)	1:6.5	1:12.9	16 (1.9)
**Kandal** (11,81002; 105,03358)	1219	237	135	742	258 (79)	132	126	36 (22)	1:2.9	1:20.6	10 (1.4)
**Sla** (11,80057; 105,0129)	391	80	137	579	46 (96)	30	16	28 (15)	1:12.6	1:20.7	10 (1.7)
**Total**	**10244**	**1908**	**1723**	**8404**	**2205 (92)**	**1001**	**1204**	**440 (385)**	**1:3.8**	**1:19.1**	**196 (2.3)**

S1.2_Table: Interviewed population, dog and cat population, ratios and bite incidence, Battambang province, Cambodia, 2018

Village Names (rural/peri-urban) (GPS coord)	Population size	Total number of Households (HH)	Number of interviewed HH	Size of the interviewed population	Number of recorded dogs during S1 (%vaccinated)	Number of males	Number of females	Number of recorded cats during S1 (%vaccinated)	Observed dog to human Ratio	Observed cat to human Ratio	Number of bite events in the previous year (per 100 people included in the survey)
**Cheu Teal (p)** (13,02798;103,17435)	1691	285	199	991	161 (80)	78	83	83 (79)	1:6.2	1:11.9	22 (2.2)
**Khna (p)** (13,02142;103,16132)	1053	216	161	764	189 (88)	102	87	65 (53)	1:4	1:11.7	24 (3.1)
**Anna Chith (p)** (13,01084;103,15753)	1523	309	190	930	230 (92)	136	94	99 (74)	1:4	1:9.4	38 (4.1)
**Svay Prokeab (p)** (12,9941;103,14127)	1076	247	156	764	216 (78	106	110	77(42)	1:3.5	1:9.9	18 (2.4)
**Kampang Lech (p)** (12,94072;103,14199)	551	151	76	324	68 (9061)	41	27	49 (35)	1:4.8	1:6.9	16 (4.9)
**Snoeng Lech (r)** (12,96728;103,05043)	1522	226	191	880	183 (82)	107	76	80 (68)	1:4.8	1:11	21 (2.4)
**Snoeng Keut (r)** (12,95683;103,08099)	2162	374	309	1451	507 (76)	251	256	155 (102)	1:2.9	1:9.4	59 (4.1)
**Preah Sre (r)** (12,96008;103,03881)	1677	290	209	983	418 (64)	217	201	107 (82)	1:2.4	1:9.2	30 (3.1)
**Rom Chey (r)** (12,92907;103,00783)	1805	302	224	1050	337 (76)	193	144	129 (95)	1:3.1	1:8.1	41 (3.9)
**Boeng Prey (r)** (12,99291;102,92586)	2262	500	367	1660	701 (75)	348	353	199 (142)	1:2.4	1:8.3	35 (3.3)
**Total**	**13872**	**2900**	**2082**	**9797**	**3010 (77)**	**1579**	**1431**	**1043 (765)**	**1:3.3**	**1:9.4**	**304 (3.1)**

S1.3_Table: Results of the Welch two sample test comparing the mean age of each village dog population to the global population (n=2185), Kandal province, Cambodia

Villages	Number of dogs	Estimated age mean	Welch test p value
Ta Koat Lech	210	18.3	3.10^{-10}
Ta Koat Kaeut	377	24.3	3.10^{-14}
Chey Touch	304	30.5	0.04
Chey Loas	138	23.6	0.08
Preak Thmei	449	18.8	3.10^{-14}
Svay Romiet	127	36.6	0.03
Chheu Teal	149	36	8.10^{-3}
Preak Ta Baen	125	33.4	6.10^{-4}
Kandal	260	29.7	0.18
Sla	46	39.8	0.14

S1.4_Table: Distribution of missing dogs during S2, per village, in Kandal and Battambang provinces

	Village names	Number of dogs included in the survival analysis	Number of missing dogs (%)	Number of missing males (%)
**Kandal**	Ta Koat Lech	210	130 (62.2)	60 (46.1)
	Ta Koat Kaeut	377	192 (50.9)	88 (45.8)
	Chey Touch	304	153 (50.3)	80 (52.3)
	Chey Loas	138	76 (55.1)	35 (46.1)
	Preak Thmei	449	301 (67.0)	123 (40.9)
	Svay Romiet	127	51 (40.2)	26 (51)
	Chheu Teal	149	52 (34.9)	30 (57.7)
	Preak TaBaen	125	72 (57.6)	31 (43.1)
	Kandal	260	129 (80.1)	52 (40.3)
	Sla	46	16 (34.8)	12 (75)
	**Total**	**2185**	**1172 (53.6)**	**537 (45.8)**
**Battambang**	Chheu Teal	107	20 (18.7)	10 (50)
	Khna Preah	125	37 (29.6)	22 (59.5)
	Anna Chit	142	37 (26.0)	20 (54)
	Svay Prakeab	151	44 (29.1)	22 (50)
	Kampang Lech	57	17 (29.8)	9 (52.9)
	Snoeng Lech	135	26 (19.2)	18 (69.2)
	Snoeng Keut	278	84 (30.2)	47 (56.0)
	Preah Sre	212	81 (38.2)	40 (49.4)
	Rum Chey	154	55 (35.7)	26 (47.3)
	Boeng Prey	312	103 (33.0)	49 (47.6)
	**Total**	**1673**	**504 (30.1)**	**265 (52.6)**

S1.5_Table: Estimated survival rates (Kaplan-Meier estimates) of the studied dog populations, Kandal and Battambang provinces, Cambodia 2017-2018

	**Kandal province (n=2185)**										**Battambang province (n=1673)**		
**Age (months)**	**Total population** (95%CI)	**Ta Koat Lech** (95%CI)	**Ta Koat Kaeut** (95%CI)	**Chey Touch** (95%CI)	**Chey Loas** (95%CI)	**Preak Thmei** (95%CI)	**Svay Romiet** (95%CI)	**Chheu Teal** (95%CI)	**Preak TaBaen** (95%CI)	**Kandal** (95%CI)	**Sla** (95%CI)	**Total population** (95%CI)	**Rural zone** (95%CI)	**Peri-urban zone** (95%CI)
**6**	0.73<br>(0.71- 0.75)	0.62<br>(0.56- 0.69)	0.71<br>(0.66-0.76)	0.77<br>(0.72-0.82)	0.78<br>(0.71-0.85)	0.60<br>(0.56-0.65)	0.84<br>(0.78-0.91)	0.82<br>(0.77-0.89)	Na	0.79<br>(0.74- 0.85)	Na	0.76<br>(0.74-0.78)	0.74<br>(0.71-0.76)	0.81<br>(0.77-0.85)
**12**	0.62<br>(0.61- 0.64)	0.53<br>(0.46- 0.60)	0.62<br>(0.58-0.68)	0.68<br>(0.64-0.74)	0.62<br>(0.54-0.71)	0.43<br>(0.39-0.48)	0.76<br>(0.69-0.84)	0.74<br>(0.67-0.81)	0.78<br>(0.71- 0.86)	0.70<br>(0.64-0.76)	0.79<br>(0.68-0.92)	0.57<br>(0.55-0.59)	0.55<br>(0.53-0.58)	0.61<br>(0.56-0.66)
**24**	0.52<br>(0.50- 0.55)	0.40<br>(0.34-0.48)	0.54<br>(0.49-0.60)	0.60<br>(0.54-0.66)	0.47<br>(0.39-0.58)	0.34<br>(0.30-0.39)	0.68<br>(0.60-0.77)	0.67<br>(0.59-0.75)	0.68<br>(0.59- 0.77)	0.58<br>(0.52-0.65)	0.71<br>(0.58-0.86)	0.34<br>(0.32-0.37)	0.33<br>(0.31-0.36)	0.38<br>(0.34-0.47)
**36**	0.46<br>(0.43-0.48)	0.33<br>(0.27- 0.42)	0.47<br>(0.42-0.53)	0.52<br>(0.46-0.58)	0.40<br>(0.32-0.51)	0.28<br>(0.23-0.33)	0.63<br>(0.54-0.73)	0.60<br>(0.52-0.69)	0.60<br>(0.51-0.71)	0.51<br>(0.44-0.58)	0.64<br>(0.50-0.82)	0.22<br>(0.20-0.24)	0.20<br>(0.18-0.22)	0.28<br>(0.24-0.32)
**48**	0.41<br>(0.39-0.44)	0.29<br>(0.22-0.38)	0.42<br>(0.36-0.49)	0.48<br>(0.42-0.55)	Na	0.24<br>(0.20-0.30)	0.61<br>(0.53-0.72)	0.52<br>(0.43-0.62)	0.53<br>(0.44-0.65)	0.45<br>(0.39-0.53)	Na	0.17<br>(0.15-0.19)	0.15<br>(0.13-0.17)	0.22<br>(0.19-0.27)
**60**	0.36<br>(0.33-0.39)	0.22<br>(0.15-0.32)	0.36<br>(0.29-0.43)	0.43<br>(0.36-0.50)	0.34<br>(0.25-0.47)	0.21<br>(0.17-0.27)	0.59<br>(0.50-0.70)	0.45<br>(0.36-0.56)	0.47<br>(0.37-0.60)	0.38<br>(0.31-0.47)	0.54<br>(0.39-0.76)	0.11<br>(0.09-0.13)	0.10<br>(0.08-0.11)	0.15<br>(0.12-0.19)
**72**	0.34<br>(0.31-0.36)	0.19<br>(0.12-0.31)	0.34<br>(0.28-0.42)	0.40<br>(0.33-0.48)	0.30<br>(0.20-0.45)	0.20<br>(0.16-0.26)	0.53<br>(0.42-0.67)	0.41<br>(0.32-0.52)	0.44<br>(0.33-0.58)	0.37<br>(0.29-0.46)	Na	0.08<br>(0.07-0.09)	0.07<br>(0.06-0.09)	0.11<br>(0.09-0.15)
**84**	Na	Na	0.32<br>(0.25-0.41)	0.36<br>(0.28-0.45)	Na	0.17<br>(0.12-0.23)	0.49<br>(0.38-0.65)	0.36<br>(0.26-0.49)	Na	Na	Na	0.06<br>(0.05-0.07)	0.05<br>(0.04-0.06)	0.09<br>(0.06-0.12)
**96**	0.29<br>(0.26-0.32)	Na	0.294<br>(0.22-0.40)	0.34<br>(0.26-0.43)	Na	Na	Na	0.33<br>(0.23-0.47)	0.38<br>(0.25-0.57)	0.33<br>(0.25- 0.43)	Na	0.04<br>(0.03-0.05)	0.04<br>(0.03-0.05)	0.06<br>(0.038-0.08)
**108**	0.29<br>(0.25-0.32)	Na	Na	0.31<br>(0.23-0.42)	Na	Na	Na	Na	Na	Na	Na	0.04<br>(0.03-0.05)	0.03<br>(0.02-0.04)	0.05<br>(0.03-0.08)
**120**	0.24<br>(0.20-0.28)	Na	Na	0.25<br>(0.16-0.38)	0.08<br>(0.01-0.43)	0.14<br>(0.09-0.21)	0.39<br>(0.26-0.59)	Na	0.31<br>(0.18-0.54)	0.27<br>(0.18-0.40)	Na	0.01<br>(0.009-0.02)	0.01<br>(0.007-0.02)	0.02<br>(0.008-0.034)
**144**	0.20<br>(0.10-0.25)	Na	0.20<br>(0.08-0.46)	Na	Na	0.09<br>(0.04-0.23)	0.30<br>(0.15-0.60)	Na	Na	0.18<br>(0.09-0.36)	Na	0.007<br>(0.004-0.01)	0.007<br>(0.004- 0.013)	0.005<br>(0.0008-0.019)
**156**	0.17<br>(0.13-0.23)	Na	Na	0.15<br>(0.06-0.34)	Na	Na	Na	Na	Na	Na	Na	0.004<br>(0.002-0.009)	0.006<br>(0.003-0.0112)	0.0008<br>(0.003-0.0112)
**168**	0.16<br>(0.11-0.22)	Na	Na	0.10<br>(0.03-0.32)	Na	Na	Na	Na	Na	Na	Na	0.0006<br>(0.00008-0.004)	Na	Na
**180**	0.12<br>(0.07-0.19)	Na	Na	0.05<br>(0.01-0.30)	Na	Na	Na	Na	Na	Na	Na	Na	0.0008<br>(0.00011- 0.006)	Na
**204**	Na	Na	Na	Na	Na	Na	Na	Na	Na	Na	Na	0.00	Na	Na

S1.6_Table: Dog ownership characteristics in Kandal province, Cambodia 2017

	Sex of the head of the family		Age class of the head of the family						Number of bite events experienced in the family in the previous year				Nb of children <15yrs										
**Number of owned dogs**	**M**	**F**	**<30**	**30-40**	**40-50**	**50-60**	**60-70**	**>70**	**0**	**1**	**2**	**3**	**0**	**1**	**2**	**3**	**4**	**5**	**6**	**7**	**8**	**9**	**15**
**0**	295	411	75	211	129	131	95	65	641	54	6	0	230	211	179	70	12	3	1	0	0	0	0
**1**	201	225	53	105	95	87	57	30	368	43	5	1	99	126	125	49	18	7	0	2	0	0	1
**2**	118	185	21	76	72	64	44	26	277	24	4	0	62	109	81	35	10	5	0	0	1	0	0
**3**	63	86	17	32	28	39	21	12	133	12	4	0	27	47	43	20	7	2	1	1	0	1	0
**4**	48	26	6	20	16	18	10	3	63	8	2	0	8	16	22	18	7	2	0	0	0	0	0
**5**	12	12	2	7	5	5	4	1	21	3	0	0	4	11	4	3	1	1	0	0	0	0	0
**6**	10	7	1	3	3	2	5	3	16	2	0	0	3	5	7	0	2	0	0	0	0	0	0
**7**	4	5	1	4	2	1	0	1	8	1	0	0	1	2	6	0	0	0	0	0	0	0	0
**8**	4	4	1	3	3	0	1	0	6	2	0	0	1	4	3	0	0	0	0	0	0	0	0
**9**	2	1	1	2	0	0	0	0	2	1	0	0	0	1	0	2	0	0	0	0	0	0	0
**10**	2	1	0	1	1	1	0	0	2	1	0	0	0	0	2	0	1	0	0	0	0	0	0
**11**	0	0	0	0	0	0	0	0	0	0	0	0	0	0	0	0	0	0	0	0	0	0	0
**12**	0	0	0	0	0	0	0	0	0	0	0	0	0	0	0	0	0	0	0	0	0	0	0
**13**	1	0	0	0	0	1	0	0	1	0	0	0	1	0	0	0	0	0	0	0	0	0	0
**14**	1	0	0	0	0	1	0	0	1	0	0	0	0	0	0	1	0	0	0	0	0	0	0

S1.7_Table (a): Dog ownership characteristics in Battambang province, Cambodia 2018

	Sex the head of the family		Age class of the head of the family						Number of bite events experienced in the family in the previous year				Nb of children <15yrs								
**Nb of owned dogs**	**M**	**F**	**<30**	**30-40**	**40-50**	**50-60**	**60-70**	**<70**	**0**	**1**	**2**	**3**	**0**	**1**	**2**	**3**	**4**	**5**	**6**	**7**	**8**
**0**	250	573	120	196	136	165	126	80	719	87	14	2	229	232	220	90	32	15	3	1	0
**1**	167	305	72	118	75	102	63	42	418	47	6	1	123	133	128	48	29	6	4	0	1
**2**	126	217	38	76	74	90	45	20	290	48	5	0	83	104	94	42	17	2	1	0	0
**3**	96	127	27	42	43	59	31	21	199	21	3	0	50	67	54	33	10	7	2	0	0
**4**	43	53	10	21	19	26	12	8	82	12	1	1	18	27	25	20	5	1	0	0	0
**5**	23	31	5	8	15	15	7	4	51	3	0	0	19	15	9	7	3	1	0	0	0
**6**	11	22	3	10	6	10	3	1	26	6	1	0	7	9	6	7	2	2	0	0	0
**7**	8	6	0	4	3	4	2	1	12	2	0	0	2	5	3	4	0	0	0	0	0
**8**	5	5	1	4	1	1	3	0	10	0	0	0	1	2	2	3	2	0	0	0	0
**9**	3	2	0	2	1	1	1	0	4	1	0	0	1	1	2	1	0	0	0	0	0
**10**	2	0	0	0	1	0	0	0	0	0	2	0	2	0	0	0	0	0	0	0	0
**11**	1	0	0	0	0	0	0	1	1	0	0	0	0	0	1	0	0	0	0	0	0
**12**	1	1	0	1	1	0	0	0	2	0	0	0	1	1	0	0	0	0	0	0	0
**13**	1	2	0	2	1	0	0	0	3	0	0	0	0	0	2	1	0	0	0	0	0
**14**	1	0	0	0	1	0	0	0	0	1	0	0	1	0	0	0	0	0	0	0	0

S1.8_Table S(b): Dog ownership characteristics in Battambang province, Cambodia 2018

	Occupation of the head of the family								Zone	
**Nb of owned dogs**	**Farmer**	**Traders**	**Unemployed**	**Industry**	**Governmental institution**	**Mechanic**	**Doctors**	**NGO's**	**Rural**	**Urban**
**0**	483	163	103	45	23	1	3	1	512	310
**1**	351	53	30	15	19	3	1	0	332	140
**2**	260	26	26	9	9	3	0	0	252	91
**3**	164	20	20	7	10	1	1	0	178	45
**4**	76	8	7	3	2	0	0	0	74	22
**5**	38	7	6	1	2	0	0	0	46	8
**6**	26	1	4	2	0	0	0	0	30	3
**7**	13	1	0	0	0	0	0	0	11	3
**8**	4	2	1	2	1	0	0	0	7	3
**9**	4	0	0	1	0	0	0	0	5	0
**10**	1	0	0	0	1	0	0	0	2	0
**11**	1	0	0	0	0	0	0	0	1	0
**12**	2	0	0	0	0	0	0	0	2	0
**13**	3	0	0	0	0	0	0	0	3	0
**14**	1	0	0	0	0	0	0	0	1	0

S1.9_Table: Dog functions, and dog population management in Kandal and Battambang provinces, Cambodia, 2017-2018

		**Function**				**Consumption**		**Confinement**		**Trade**		**Puppies management**				**Prefered gender**			**Dog origins**			
		**Family member**	**Business**	**Guard**	**Hunt**	**Yes**	**No**	**Yes**	**No**	**Yes**	**No**	**Keep and raise**	**Sale**	**Cull**	**Aband**	**Male**	**Female**	**Both**	**Family**	**Gift**	**Found**	**Purchased**
**Kandal**	**All interviewed families (1723)**	Na	Na	Na	Na	404	1319	Na	Na	1366	57	Na	Na	Na	Na	Na	Na	Na	Na	Na	Na	Na
	**Families with dogs (n=1017)**	208	14	793	2	292	725	6	1011	280	737	1000	13	2	2	222	179	616	Na	Na	Na	Na
	**Dogs (n=2205)**	Na	Na	Na	Na	Na	Na	Na	Na	Na	Na	Na	Na	Na	Na	Na	Na	Na	966	678	53	508
**Battambang**	**All interviewed families (n =2082)**	Na	Na	Na	Na	1221	871	Na	Na	279	1803	Na	Na	Na	Na	Na	Na	Na	Na	Na	Na	Na
	**Families with dogs (n=1260)**	18	3	1237	2	812	448	13	1247	230	1030	1254	5	1	0	308	55	897	Na	Na	Na	Na
	**Dogs (n=3010)**	Na	Na	Na	Na	Na	Na	Na	Na	Na	Na	Na	Na	Na	Na	Na	Na	Na	1511	1360	97	42
