# Supplementary figures and images for: Large scale dog population demography, dog management and bite risk factors analysis: A crucial step towards rabies control in Cambodia
Source: PLoS One. 2021 Jul 8;16(7):e0254192. doi: 10.1371/journal.pone.0254192 (PMC8266089; doi:10.1371/journal.pone.0254192)

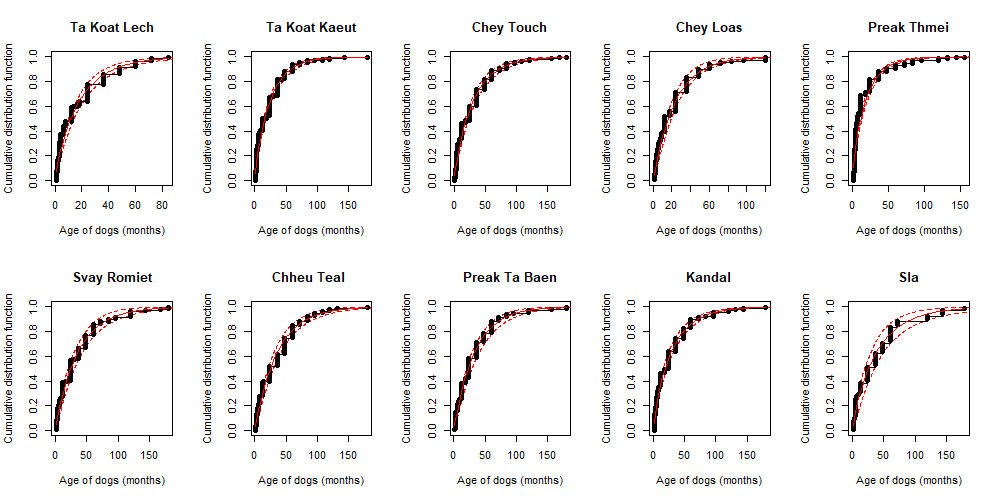

Supplement: S1 Fig — (TIFF) [file pone.0254192.s004.tiff]

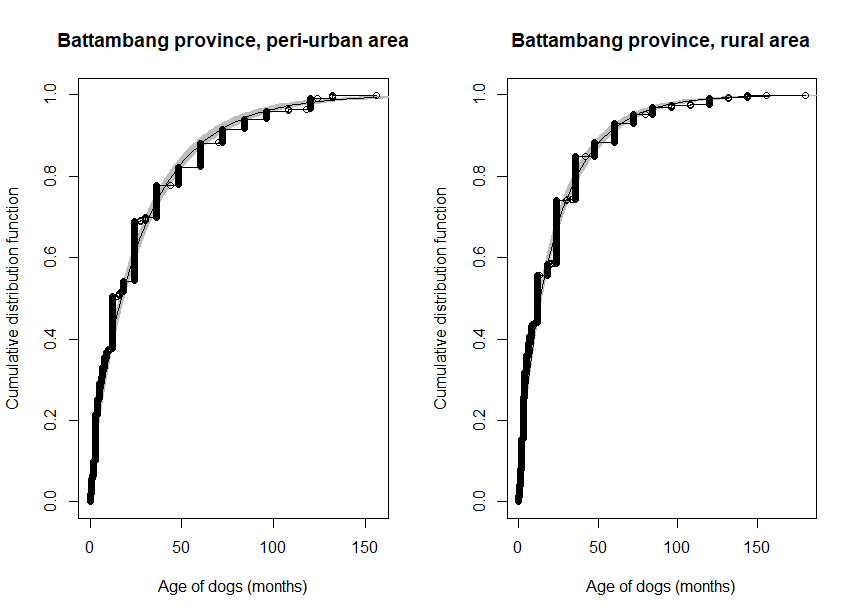

Supplement: S2 Fig — (TIFF) [file pone.0254192.s005.tiff]
